# Executive functions in adults born small for gestational age at term: a prospective cohort study

**DOI:** 10.1038/s41598-025-86241-2

**Published:** 2025-01-29

**Authors:** Mariell Nordgård, Martine Reitan Udnæs, Kristina Anna Djupvik Aakvik, Silje Dahl Benum, Sigrid Hegna Ingvaldsen, Siri Weider, Kari Anne I. Evensen

**Affiliations:** 1 Faculty of Medicine and Health Sciences, Norwegian University of Science and Technology, Trondheim, Norway; 2 Department of Clinical and Molecular Medicine, Norwegian University of Science and Technology, Trondheim, Norway; 3 Department of Psychology, Norwegian University of Science and Technology, Trondheim, Norway; 4 Children’s Clinic, St. Olavs Hospital, Trondheim University Hospital, Trondheim, Norway; 5 Department of Rehabilitation Science and Health Technology, Oslo Metropolitan University, Oslo, Norway

**Keywords:** Health care, Medical research

## Abstract

There are mixed findings regarding executive functioning in individuals born small for gestational age (SGA) at term and associations between performance-based and self-reported executive functions have yet to be examined in adults. In a prospective cohort study, 56 SGA and 68 non-SGA control participants were assessed at 32 years using the performance-based Trail Making Test (TMT) and the self-report questionnaire Behavior Rating Inventory of Executive Function – Adult Version (BRIEF-A). The SGA group used 1.6 (95% confidence interval [CI] 0.1–3.1) to 3.9 (95% CI 1.2–6.5) seconds more on TMT conditions 1 and 3, indicating more difficulties with visual scanning and letter sequencing than controls. On BRIEF-A, the SGA group scored 1.5 (95% CI 0.2–2.8) points lower on the clinical scale Emotional Control. Correlation coefficients for the association between TMT and BRIEF-A scores ranged from -0.295 (*p* = 0.065) to 0.345 (*p* = 0.029) in the SGA group. In conclusion, we found that 32-year-old adults born SGA at term showed poorer performance on some executive function tasks than controls. However, they reported similar executive functioning in everyday life, except for emotional control. The associations between performance-based and self-report assessments of executive functions were weak.

**Supplementary Information:**

The online version contains supplementary material available at 10.1038/s41598-025-86241-2.

## Introduction

Being born small for gestational age (SGA; birth weight < 10th percentile for gestational age) is associated with multiple adverse health outcomes later in life. Adults born SGA at term have a higher risk of obesity, type 2 diabetes and cardiovascular disease^1^, poorer mental health^2,3^, and psychiatric disorders such as anxiety, attention deficit hyperactivity disorder (ADHD) and mood disorders^4,5^. Multiple studies have also demonstrated structural underdevelopment in the brains of both newborns and adults who were born both preterm and at term with low birth weight^6–8^. In terms of cognitive functions, some studies suggest that individuals born SGA experience more cognitive difficulties and have a lower intelligence quotient (IQ) than their non-SGA peers^9,10^. However, other studies have not identified differences in cognitive development or functioning between SGA and non-SGA individuals^11,12^.

Several studies have examined executive functions in individuals born with low birth weight. However, most of this research has been performed in individuals born preterm and/or with very low birth weight (VLBW). These studies report impaired functions across various executive domains among these individuals compared with term-born controls^13–16^. Executive dysfunctions, especially in neurocognitive tests that measure performance speed, have also been found in young adults born SGA at term^17–19^. In contrast, other studies show that individuals born SGA late preterm or at term perform similarly as their non-SGA term-born peers in most executive functions^20,21^.

Most of the research on executive functions relies solely on performance-based measures, with few studies exploring the association between performance-based and self-reported executive functions. However, as self-reports are used to evaluate executive functions in a range of clinical settings^22^, the relationship between the two measures is highly relevant. Only one Norwegian study by Sølsnes and colleagues^23^ and one Finnish study by Heinonen and colleagues^24^ have investigated this objective in low-birth-weight populations. Both studies found that adults born with VLBW, especially those born SGA, scored lower on performance-based tests assessing executive functions compared with the non-SGA control group. However, neither study found differences in self-reported executive functions between the groups, indicating that the individuals born with VLBW did not perceive executive dysfunctions themselves. Furthermore, associations between the performance-based test scores and the self-reports were weaker in the VLBW group compared with the control group.

No studies have examined executive functions in individuals born SGA at term beyond the third decade of life. There is evidence to suggest that the prefrontal cortex, and consequently executive functions, continues to develop throughout the twenties before stabilizing in the thirties^25^. We aimed to examine if executive function was poorer in adults born SGA at term compared with non-SGA term-born controls and investigate the association between performance-based and self-reported measures of executive function. Based on the previous findings in adult populations born preterm, we hypothesized that adults born SGA would exhibit poorer executive function on performance-based tests, but that they would have similar levels of executive functioning as controls by self-report. Therefore, we expected that the correlation between performance-based and self-reported measures of executive functions would be weaker in the SGA group compared with the control group.

## Methods

### Study design

This study is part of the NTNU Low Birth Weight in a Lifetime Perspective (NTNU LBW Life) study, where two groups of adults born at term (gestational age ≥ 37 weeks) between 1986 and 1988 have been followed prospectively from birth. One group was born SGA (birth weight < 10th percentile), while the other group was born non-SGA (birth weight ≥ 10th percentile). The mothers of the participants were initially recruited from a multicenter study of pregnant women aimed to assess causes and consequences of intrauterine growth restriction^26,27^. They were all carrying a singleton and had given birth at least once before. All participants were born to women residing in the same geographical region.

The participants have been examined at several time points from early childhood to adulthood. In the present study, data from the 32-year follow-up were used. Data were collected from September 2019 to January 2021. Examiners were blinded to group status. In addition to examination of executive functions, the participants underwent assessments of motor and visual function, physical fitness^28^, physical activity^29^ and health-related quality of life^30^.

### Study population

**Fig. 1:**
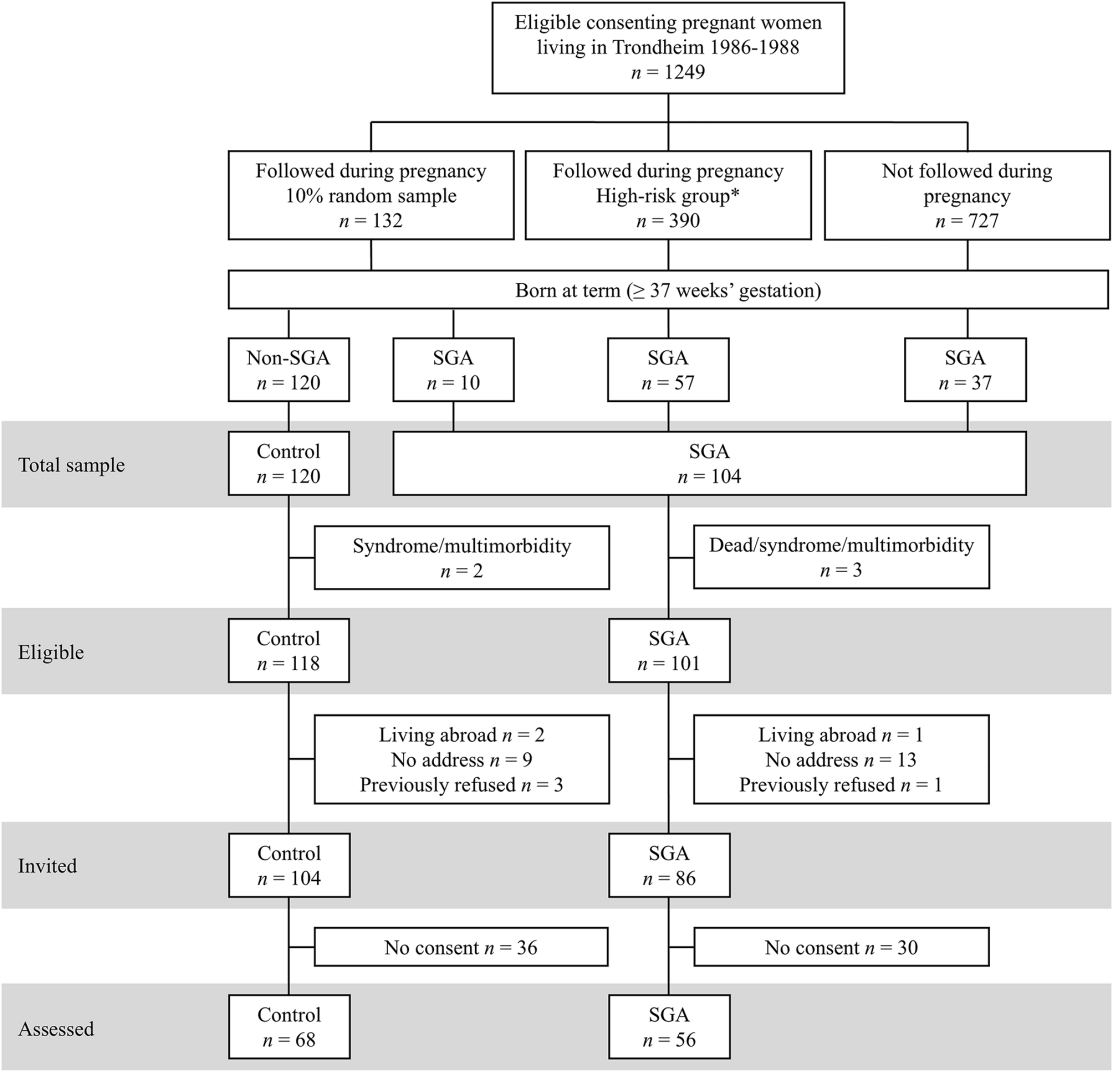
Flow of participants at 32 years of age. *The high-risk group consisted of women with one or more of the following risk factors: previous low birth weight child, low pre-pregnancy weight (< 50 kg), previous perinatal death, chronic maternal disease and smoking. *SGA *small for gestational age

A total of 1249 women in Trondheim, Norway, consented to participate in the initial multicenter study (Fig. 1). The women were split into three groups. First, a random 10% sample of women were selected for follow-up during pregnancy (*n* = 132), regardless of risk factors. Next, a sample of women at high risk of delivering an SGA infant were selected for follow-up (*n* = 390). These women exhibited at least one of five predetermined risk factors: a previous child with low birth weight or perinatal death, a pre-pregnancy weight < 50 kg, chronic maternal disease (i.e. hypertension, heart or renal disease) or smoking at the time of conception. The remaining pregnant women included in the multicenter study from the total eligible population did not receive additional follow-up throughout pregnancy (*n* = 727). All infants with a birth weight < 10th percentile at term born to women in the random sample, the high-risk sample or the sample not followed in pregnancy were included in the SGA group (*n* = 104) and infants born to women in the random sample with a birth weight ≥ 10th percentile formed the control group (*n* = 120).

#### SGA group

The SGA group included 104 individuals with a birth weight < 10th percentile adjusted for gestational age, sex and parity, according to a reference standard presented by the Norwegian Medical Birth Registry^31^. Out of 104 SGA participants, three were excluded due to congenital syndromes, multimorbidity or death before follow-up. At the 32-year follow-up, 15 individuals were living abroad, could not be reached or had previously refused to participate. The remaining 86 individuals were invited to participate, but 30 of these did not consent, leaving 56 participants. In total, 56 (31 women, 25 men) adults born SGA (65% of invited and 55% of eligible) participated and were included in the current study.

#### Control group

The control group included 120 individuals born at term with a birth weight ≥ 10th percentile. Two of these were excluded due to congenital syndromes or multimorbidity. Out of the 118 eligible controls at the 32-year follow-up, 14 were living abroad, could not be reached or had previously refused to participate. Altogether, 104 participants were invited, but 36 did not consent, which left 68 participants in the control group. In total, 68 (39 women, 29 men) controls (65% of invited and 58% of eligible) participated and were included in the current study.

#### Non-participants

There were no differences in background characteristics between participants and those who did not consent to participate at the age of 32 years in either group (Additional file; Table S1).

### Background characteristics

At birth, sex, gestational age, birth weight, birth length, head circumference and maternal age were reported for all infants. Data on parental socioeconomic status (SES) was collected during follow-up assessments when the participants were 14 years and supplemented at 19 years. We used the Hollingshead Two-Factor Index of Social Position, which is based on a combination of education and occupation, ranging from 1 at the lowest to 5 at the highest^32^. When the participants were 32 years of age, their education was reported and categorized into three groups based on the International Standard Classification of Education (ISCED) levels: lower secondary or lower, intermediate and lower tertiary or higher.

### Outcome measures

#### Trail Making Test

The Trail Making Test (TMT) from the Delis-Kaplan Executive Function System (D-KEFS)^33^ provides an objective measure of executive functions. The conditions being tested are visual scanning (TMT 1), number sequencing (TMT 2), letter sequencing (TMT 3), number-letter switching (TMT 4) and motor speed (TMT 5). TMT 1–3 assess basic attention and processing speed, TMT 4 additionally requires set shifting, inhibition and working memory, and TMT 5 measures motor speed and eye-hand coordination. The participants were asked to connect circles, either empty or containing a certain value (number or letter), in a particular order. Longer time spent on the tests indicate poorer executive function. To isolate the effect of cognitive flexibility, a variable subtracting the time spent to complete the TMT 2 from the time spent to complete the TMT 4 (TMT 4-2) was calculated. We present both raw scores, i.e. time in seconds used to complete the tasks, and calculated scaled scores according to the participants’ age based on the normative sample in the manual^33^ with a mean of 10 (standard deviation [SD] 3). The TMT assessment was carried out by examiners blinded to group adherence.

#### Behavior Rating Inventory of Executive Function – Adult Version

The Behavior Rating Inventory of Executive Function – Adult Version (BRIEF-A)^34^ is a standardized self-report questionnaire for adults assessing the participants’ subjective perception of executive functioning in everyday activities over the last month. It is composed of 75 items comprising nine scales. Participants are asked how often each of the listed behaviors has been a problem during the past month and must place them on a 3-point Likert scale, with the options never, sometimes and often. The answers are summarized into three composite scores: the overall Global Executive Composite and the two index scores Behavioral Regulation Index and Metacognition Index. The Behavioral Regulation Index measures the individual’s ability to appropriately regulate emotions and behavior, and is composed of the clinical scales Inhibit, Shift, Emotional Control and Self-Monitor. The Metacognition Index measures the individual’s ability to solve problems across different contexts, and is composed of the clinical scales Initiate, Working Memory, Plan/Organize, Task Monitor and Organization of Materials. Higher scores indicate poorer executive functioning.

### Ethics

This study was conducted in accordance with the Helsinki Declaration and approved by the Regional Committee for Medical and Health Research Ethics (REK) in Central Norway (23879). The adult participants gave written informed consent. The clinical assessments were non-invasive and did not entail risk for injury or adverse events. Participants received medically relevant feedback and were referred to health services as appropriate. Data was handled securely in Services for Sensitive Data (TSD).

### Statistical analyses

Data was analyzed using IBM SPSS Statistics 29.0. Statistical significance was set at two-sided p-value below 0.05 and a confidence interval (CI) of 95% were reported when relevant. Normality was assessed by visual inspection of histograms and Q-Q plots of the residuals. Group differences in background characteristics were analyzed using the independent Student’s t-test for continuous and normally distributed data, Pearson’s Chi square for categorical variables and Mann-Whitney U Test for ordinal data. Unadjusted mean (SD) and Cohen’s d were given for the TMT conditions and BRIEF-A clinical scales and composite scores. Effect sizes were interpreted as small (0.2), medium (0.5) or large (0.8)^35^. Group differences were analyzed using linear regression with TMT and BRIEF-A scores as the dependent variable, group (SGA vs. control) as a fixed factor, and sex as a covariate. In additional analyses, parental SES was also included as a covariate. Associations between TMT and BRIEF-A scores were examined using partial correlations, adjusted for sex.

## Results

### Background characteristics

Background characteristics of the participants are presented in Table 1. The SGA participants were smaller at birth with lower birth weight, birth length, ponderal index and head circumference compared with the control participants. Maternal age at delivery was on average 2.5 years lower in the SGA group compared with the non-SGA control group. Gestational age, parental SES, age at follow-up and participants’ educational levels were similar in the two groups.

**Table 1:** Background characteristics of participants in the SGA and non-SGA control groups.

	SGA			Control			*p*-value
	*n*	Mean	(SD)	*n*	Mean	(SD)	
Gestational age (weeks)	56	39.7	(1.2)	68	39.8	(1.2)	0.454
Birth weight (g)	56	2916	(205)	68	3695	(459)	< 0.001
Birth length (cm)	49	48.5	(1.9)	65	51.1	(1.9)	< 0.001
Ponderal index (g/cm^3^)	49	2.6	(0.2)	65	2.8	(0.3)	< 0.001
Birth head circumference (cm)	50	33.9	(1.1)	64	35.4	(1.2)	< 0.001
Maternal age at delivery (years)	50	28.2	(3.2)	66	30.7	(4.3)	< 0.001
Parental SES	47	3.5	(1.2)	57	3.7	(1.1)	0.439
Age at follow-up (years)	48	32.5	(0.6)	61	32.6	(0.5)	0.841
		***n***	**(%)**		***n***	**(%)**	
Female	56	31	(55.4)	68	39	(57.4)	0.823
Education at follow-up	56			68			0.246
Lower secondary or lower		2	(3.6)		0	(0.0)	
Intermediate		22	(39.3)		23	(33.8)	
Lower tertiary or higher		32	(57.1)		45	(66.2)	

*SD* standard deviation, *SES* socioeconomic status, *SGA* small for gestational age.

### Performance-based executive function

Table 2 presents TMT raw scores (seconds) and scaled scores in the two groups. Unadjusted effect sizes ranged from 0.16 for the difference between number-letter and number sequencing (TMT 4-2) to 0.59 for letter sequencing (TMT 3). Adjusted for sex, the SGA group used 1.6 (95% CI 0.1–3.1) seconds more at visual scanning (TMT 1) and 3.9 (95% CI 1.2–6.5) seconds more at TMT 3. These differences were also reflected by lower scaled scores for these conditions.

**Table 2:** Performance-based executive function measured by the Trail Making Test (TMT) in the SGA and non-SGA control groups at 32 years of age.

	SGA			Control			Effect size	Mean difference (95% CI)^a^		*p*-value	Mean difference (95% CI)^b^		*p*-value	Mean difference (95% CI)^c^		*p*-value
	*n*	Mean	(SD)	*n*	Mean	(SD)	(Cohen’s d)									
Raw scores (sec)																
TMT 1	46	18.5	(4.6)	61	16.7	(3.5)	0.44	1.6	(0.1, 3.1)	0.041	1.6	(0.1, 3.2)	0.041	1.5	(-0.1, 3.1)	0.059
TMT 2	46	24.1	(7.3)	61	22.0	(6.9)	0.30	2.0	(-0.7, 4.8)	0.145	3.3	(0.3, 6.3)	0.031	3.2	(0.2, 6.2)	0.035
TMT 3	45	24.9	(7.6)	61	21.0	(5.9)	0.59	3.9	(1.2, 6.5)	0.004	3.9	(1.1, 6.7)	0.007	3.9	(1.1, 6.8)	0.008
TMT 4	45	65.6	(22.3)	61	60.6	(18.6)	0.25	4.6	(-3.3, 12.5)	0.251	5.2	(-3.8, 14.1)	0.254	5.4	(-3.8, 14.7)	0.242
TMT 4-2	45	41.7	(21.8)	61	38.7	(17.4)	0.16	2.7	(-4.9, 10.2)	0.487	2.0	(-6.5, 10.6)	0.639	2.3	(-6.5, 11.1)	0.600
Scaled scores																
TMT 1	46	11.1	(2.0)	61	11.9	(1.5)	− 0.47	-0.7	(-1.4, -0.1)	0.030	-0.7	(-1.4, -0.1)	0.031	-0.7	(-1.4, -0.03)	0.040
TMT 2	46	12.1	(2.0)	61	12.6	(2.0)	− 0.26	-0.5	(-1.3, 0.3)	0.213	-0.8	(-1.6, 0.05)	0.064	-0.8	(-1.6, -0.1)	0.074
TMT 3	45	11.6	(1.9)	61	12.6	(1.5)	− 0.57	-1.0	(-1.6, -0.3)	0.006	-1.0	(-1.7, -0.2)	0.010	-1.0	(-1.7, -0.2)	0.010
TMT 4	45	10.8	(2.1)	61	11.4	(1.8)	− 0.27	-0.5	(-1.3, 0.3)	0.200	-0.5	(-1.4, 0.3)	0.217	-0.6	(-1.4, 0.3)	0.213
TMT 4-2	45	− 1.3	(2.6)	61	− 1.3	(2.1)	− 0.03	-0.1	(-1.0, 0.9)	0.915	0.2	(-0.8, 1.2)	0.681	0.2	(-0.9, 1.2)	0.749

*CI* confidence interval, *SD* standard deviation, *SES* socioeconomic status, *SGA* small for gestational age, *TMT* Trail Making Test.

^a^Adjusted for sex.

^b^Adjusted for sex among participants with parental SES (data missing on TMT 1 and TMT 2 for seven SGA and 10 control participants and on TMT 3 and TMT 4 for six SGA and 10 control participants).

^c^Adjusted for sex and parental SES (data missing on TMT 1 and TMT 2 for seven SGA and 10 control participants and on TMT 3 and TMT 4 for six SGA and 10 control participants).

When we adjusted for sex among participants with available data on parental SES, the difference in time spent at number sequencing (TMT 2) also became significant. Additional adjustment for parental SES did not change the results.

### Self-reported executive function

Clinical scales and composite scores of the BRIEF-A in the two groups are presented in Table 3. Unadjusted effect sizes ranged from 0.06 for the clinical scale Organization of Materials to 0.41 for Emotional Control. Adjusted for sex, the SGA group had 1.5 (95% CI 0.2–2.8) points higher score than the control group on Emotional Control. The difference was 1.7 (95% CI 0.2–3.1) points among participants with available data on parental SES and did not change much after adjustment for parental education. There were no other group differences in BRIEF-A clinical scales or composite scores.

**Table 3:** Self-reported executive function measured by the Behavior Rating Inventory of Executive Function – Adult Version in the SGA and non-SGA control groups at 32 years of age.

	SGA (*n* = 52)		Control (*n* = 64)		Effect size	Mean difference (95% CI)^a^		*p*-value	Mean difference (95% CI)^b^		*p*-value	Mean difference (95% CI)^c^		*p*-value
	Mean	(SD)	Mean	(SD)	(Cohen’s d)									
Inhibit	10.7	(2.8)	10.3	(2.4)	0.17	0.4	(-0.5, 1.3)	0.388	0.4	(-0.6, 1.4)	0.389	0.5	(-0.6, 1.5)	0.385
Shift	8.3	(2.4)	7.9	(2.4)	0.18	0.4	(-0.4, 1.3)	0.326	0.6	(-0.4, 1.5)	0.237	0.5	(-0.4, 1.4)	0.289
Emotional Control	14.2	(3.9)	12.8	(3.4)	0.41	1.5	(0.2, 2.8)	0.026	1.8	(0.4, 3.2)	0.011	1.7	(0.2, 3.1)	0.023
Self-Monitor	7.8	(2.0)	7.1	(1.6)	0.36	0.6	(-0.04, 1.3)	0.063	0.4	(-0.3, 1.1)	0.257	0.4	(-0.3, 1.1)	0.267
Initiate	12.5	(3.5)	11.8	(3.4)	0.20	0.7	(-0.6, 1.9)	0.307	0.7	(-0.7, 2.1)	0.346	0.7	(-0.7, 2.1)	0.332
Working Memory	11.4	(3.5)	10.5	(3.2)	0.25	0.8	(-0.4, 2.1)	0.184	0.8	(-0.5, 2.2)	0.232	0.7	(-0.7, 2.0)	0.323
Plan/Organize	14.3	(3.8)	13.5	(4.3)	0.18	0.7	(-0.8, 2.2)	0.356	0.7	(-0.9, 2.3)	0.367	0.7	(-1.0, 2.3)	0.419
Task Monitor	8.9	(2.1)	8.6	(2.4)	0.17	0.4	(-0.5, 1.2)	0.370	0.3	(-0.6, 1.1)	0.577	0.3	(-0.7, 1.2)	0.553
Organization of Materials	11.8	(2.9)	11.6	(3.3)	0.06	0.2	(-1.0, 1.3)	0.759	0.01	(-1.2, 1.2)	0.991	0.1	(-1.2, 1.3)	0.928
Behavioral Regulation Index	41.1	(9.0)	38.1	(8.2)	0.35	3.0	(-0.2, 6.2)	0.068	3.2	(-0.1, 6.5)	0.061	3.0	(-0.4, 6.5)	0.083
Metacognition Index	54.8	(12.0)	50.8	(11.0)	0.35	4.0	(-0.3, 8.2)	0.068	4.2	(-0.2, 8.7)	0.061	4.0	(-0.5, 8.6)	0.083
Global Executive Composite	99.9	(21.3)	94.1	(22.4)	0.27	5.7	(-2.4, 13.8)	0.167	5.6	(-2.9, 14.1)	0.195	5.4	(-3.4, 14.2)	0.225

*CI* confidence interval, *SD* standard deviation, *SES* socioeconomic status, *SGA* small for gestational age.

^a^Adjusted for sex.

^b^Adjusted for sex among participants with parental SES (data missing for seven SGA and 10 control participants).

^c^Adjusted for sex and parental SES (data missing for seven SGA and 10 control participants).

### Correlation between self-reported and performance-based executive functions

The correlations between BRIEF-A clinical scales and composite scores and TMT raw scores are presented in Table 4 for the SGA group and Table 5 for the control group. In the SGA group, higher scores on Emotional Control correlated with longer time spent on both TMT 4 (*r* = 0.332) and higher raw scores on TMT 4-2 (*r* = 0.345). In the control group, higher scores on the Behavioral Regulation Index correlated with longer time spent on TMT 4 (*r* = 0.273) and higher raw scores on TMT 4-2 (*r* = 0.314). Furthermore, higher scores on the clinical scales Shift, Emotional Control and Plan/Organize correlated with higher raw scores on TMT 4-2 in the control group, with correlation coefficients ranging from 0.318 to 0.276.

**Table 4:** Partial correlation between BRIEF-A clinical scales and composite scores and TMT raw scores in the SGA group at 32 years of age, adjusted for sex.

	TMT 1		TMT 2		TMT 3		TMT 4		TMT 4-2	
	*r*	*p*-value	*r*	*p*-value	*r*	*p*-value	*r*	*p*-value	*r*	*p*-value
Inhibit	-0.217	0.179	-0.208	0.199	-0.205	0.205	0.230	0.153	0.307	0.054
Shift	-0.012	0.940	0.080	0.626	-0.147	0.365	0.202	0.212	0.173	0.286
Emotional Control	0.035	0.841	-0.035	0.831	-0.247	0.125	0.332	0.036	0.345	0.029
Self-Monitor	-0.006	0.969	-0.270	0.091	-0.284	0.076	-0.007	0.965	0.092	0.571
Initiate	0.056	0.730	0.063	0.700	-0.128	0.431	0.102	0.529	0.079	0.626
Working Memory	-0.033	0.842	-0.043	0.794	-0.278	0.082	0.099	0.544	0.115	0.482
Plan/Organize	-0.064	0.695	0.000	0.998	-0.206	0.202	0.256	0.110	0.257	0.110
Task Monitor	-0.247	0.124	-0.102	0.531	-0.295	0.065	0.208	0.198	0.246	0.126
Organization of Materials	-0.013	0.937	0.039	0.813	-0.185	0.252	0.030	0.852	0.016	0.921
Behavioral Regulation Index	-0.061	0.706	-0.121	0.456	-0.264	0.100	0.255	0.112	0.300	0.060
Metacognition Index	-0.053	0.743	-0.002	0.989	-0.249	0.121	0.164	0.311	0.165	0.308
Global Executive Composite	-0.059	0.716	-0.052	0.752	-0.267	0.095	0.210	0.194	0.229	0.155

*BRIEF-A* Behavior Rating Inventory of Executive Function – Adult Version, *r* correlation coefficient, *SGA* small for gestational age, *TMT* Trail Making Test.

**Table 5:** Partial correlation between BRIEF-A clinical scales and composite scores and TMT raw scores in the non-SGA control group at 32 years of age, adjusted for sex.

	TMT 1		TMT 2		TMT 3		TMT 4		TMT 4-2	
	*r*	*p*-value	*r*	*p*-value	*r*	*p*-value	*r*	*p*-value	*r*	*p*-value
Inhibit	-0.016	0.907	0.130	0.341	0.063	0.642	0.239	0.076	0.203	0.133
Shift	-0.021	0.880	-0.123	0.366	0.024	0.861	0.250	0.063	0.318	0.017
Emotional Control	-0.033	0.810	-0.096	0.481	0.021	0.876	0.243	0.072	0.299	0.025
Self-Monitor	-0.094	0.492	-0.069	0.612	0.132	0.334	0.149	0.273	0.188	0.166
Initiate	0.014	0.918	-0.001	0.993	0.101	0.458	0.088	0.518	0.095	0.486
Working Memory	0.000	0.999	-0.073	0.592	-0.011	0.936	0.153	0.260	0.193	0.153
Plan/Organize	0.036	0.790	-0.050	0.715	0.119	0.383	0.239	0.077	0.276	0.040
Task Monitor	0.018	0.893	-0.078	0.566	0.143	0.296	0.197	0.146	0.242	0.072
Organization of Materials	0.081	0.553	0.077	0.572	0.098	0.470	0.150	0.269	0.130	0.341
Behavioral Regulation Index	-0.042	0.758	-0.054	0.695	0.059	0.666	0.273	0.042	0.314	0.018
Metacognition Index	0.035	0.800	-0.026	0.850	0.098	0.474	0.184	0.175	0.207	0.125
Global Executive Composite	0.008	0.953	-0.038	0.783	0.088	0.519	0.226	0.094	0.257	0.056

*BRIEF-A* Behavior Rating Inventory of Executive Function – Adult Version, *r* correlation coefficient, *SGA* small for gestational age, *TMT* Trail Making Test.

The correlations with TMT scaled scores were of similar magnitude (Additional files; Table S2 and S3), but significant also for the clinical scale Task Monitor and TMT 3 in the SGA group.

## Discussion

To our knowledge, this study is the first to explore both performance-based and self-reported measures of executive functions in adults born SGA at term. Overall, our results suggest that adults born SGA have poorer executive functioning in some performance-based tasks than their non-SGA peers. However, the same results were not observed for self-reported executive functions. Except for emotional control, adults born SGA did not report executive dysfunctions in everyday life. Moreover, the correlations between performance-based and self-reported executive functions were weak in both groups. These findings align with our a priori hypotheses.

The strengths of this study include the prospective cohort design, use of standardized validated methods and blinded assessment. However, a potential limitation is the use of the 10th percentile definition of SGA, which does not differentiate between genetically small infants and infants who have experienced intrauterine growth restriction (IUGR). Moreover, loss to follow-up is an issue in any long-term study. The relatively small sample size may reduce the ability to detect differences between the groups and associations within the groups. Non-significant differences and associations should therefore be interpreted with caution. A small sample size may further limit the generalizability of the study. However, there were no differences in available background characteristics of participants and non-participants, indicating that our sample was representative of the initial study population.

Both the TMT and the BRIEF-A are widely used for clinical assessment and research to assess various executive processes. The TMT is sensitive to executive dysfunction, intuitive and easy to understand, and has a short administration^36^. However, it does not discriminate well between the multiple functions needed to carry it out, such as motor and visual functions, attention, and executive functions. Moreover, the TMT places significant demands on performance speed. Also, test-retest reliability indices are only moderate for ages 20–49 years, ranging from 0.36 at the lowest for TMT 3 to 0.55 for TMT 1^33^. As performance-based measures of executive functions may not accurately reflect the application of executive function skills in an everyday environment, we also used the BRIEF-A, which may offer a better portrayal of the individuals’ self-perceived executive strengths and weaknesses^37^. However, self-report measures are vulnerable to response biases, where the results can be affected by social desirability and/or extreme responses, knowledge of the individual and by cultural factors^38^. Nevertheless, the test-retest reliability of BRIEF-A is high, ranging from 0.82 at lowest for the clinical scale Plan/Organize to 0.94 at highest for the Global Executive Composite in adults aged 18–90 years in the original standardization sample^37^.

As hypothesized, the SGA group performed poorer on some of the performance-based measures. Significant group differences were of moderate effect sizes ranging from 0.30 to 0.59. Our findings that the SGA group used longer time than the control group on two conditions of the TMT aligns with previous findings in the same SGA population. Østgård and colleagues^18^ administered a comprehensive battery of neuropsychological tests to assess the executive functions when the participants in the NTNU LBW Life study were 19–20 years old. They found that the SGA group performed significantly poorer than the control group on the TMT 1, TMT 2 and TMT 3 with effect sizes ranging from -0.40 to -0.61. However, the SGA group performed as well as or better than the control group on other tests of executive functions, such as the Wisconsin Card Sorting Test and Tower test, where performance speed is less important. In a study by Weider and colleagues^19^, examining this population at 26 years of age, the SGA group performed slower than the controls on all TMT conditions. Moreover, our results are supported by a study by Suffren and colleagues^17^ reporting lower performances on tests assessing attention and executive functions in adults born SGA at 20 years of age.

Despite of the SGA group performing poorer than the controls on some of the TMT conditions, they reported similar levels of executive functioning on the BRIEF-A, except for the clinical scale Emotional Control. As such, the correlation between the performance-based TMT and the self-reported BRIEF-A was weak. Similar findings were reported by both Sølsnes and colleagues^23^ and Heinonen and colleagues^24^, even though they examined preterm populations. They found that adults born with VLBW exhibited more executive dysfunctions in performance-based tests compared with controls. However, they rated their executive functioning comparably to the control group on the BRIEF-A. Both studies reported weak correlations between performance-based measures and self-reported measures of executive functions. Results from our study are consistent with these findings.

Although the TMT results in both groups were close to the population norm, the poorer performance in the SGA group on TMT conditions 1 and 3 suggests some difficulties with visual scanning and letter sequencing. Visual functions were examined by Lindqvist and colleagues in the same SGA cohort in adolescence^39,40^, indicating no visual impairments that can explain the results. Alternative explanations may be related to brain development in areas and networks needed for higher-order cognitive processes. Several studies, performed both postmortem and in vivo, show that being born SGA with and without documented IUGR leads to a reduction in total brain volume^6^. An in vivo study on preterm infants born with IUGR using quantitative volumetric magnetic resonance imaging, showed pronounced reductions in cerebral cortical gray matter^7^. The degree of volume reduction correlated with both head circumference and functional outcome at term, most prominently attention. In our cohort, Eikenes and colleagues^8^ showed that being born SGA leads to microstructural changes in white matter in adulthood. The authors speculated that these changes are caused by a reduced number of axons and myelination present from birth, causing differences in the development of white matter. The slower performance on some of the TMT conditions in the SGA compared with the control group may be related to subtle changes in the white matter.

Poorer emotional control in the SGA group could be related to functional problems analogous to those seen in ADHD and mood disorders. The Emotional Control scale measures the impact of executive functions within the emotional domain and the ability to regulate emotional responses^34^. In our cohort, Lund and colleagues^4^ found higher psychiatric morbidity in the SGA group compared with the control group at 19 years of age, the most prevalent diagnoses being anxiety and ADHD. Additionally, the SGA group reported more inattention and hyperactivity. Similar results were found in the SGA group at 26 years of age with higher overall prevalence of psychiatric disorders, especially anxiety and mood disorders^5^. If these disorders are still present in our SGA group, it may explain the lower self-reported emotional control.

Prior research has highlighted a generally weak correlation between performance-based and self-reported measures of the same construct^41^. Whereas performance-based measures provide an indication of processing efficiency, the self-report measures may provide an indication of individual goal pursuit^42^. A meta-analysis found very small to modest associations between performance-based and self-report measures, indicating that the two measures of executive function assess different aspects^42^. Consequently, this could explain the weak association between performance-based and self-report measures in our study. Thus, it is important to be aware that the two methods cannot be used interchangeably in research or in clinical settings. It is also noteworthy that the adults born SGA with poorer performance on tests measuring executive functions did not experience this as a hinder in their everyday life.

Our results suggest that some of the difficulties in executive functions identified in adolescence and young adulthood in the same study population are still present in mid-adulthood. With SGA contributing to a sizable proportion of the population (10% per definition), the long-term effects of low birth weight even if born at term are important and could have a potentially large influence on public health and societal welfare. Executive dysfunctions have been associated with poorer academic performance^16^ and an increased prevalence of psychiatric disorders^43,44^. Weaker school performances and the need for special education can lead to lower levels of education, limited professional attainment and lower income. Additionally, other factors such as marital status, mental health and satisfaction with life might be adversely affected. Our findings indicate that term-born SGA adults may have some challenges in performance-based executive functioning compared with their peers, even though they did not report poorer executive functioning in everyday life. Further research on the long-term effects of low birth weight even if born at term is crucial to enhance our understanding of this issue and ensure the well-being of this population.

## Conclusion

In this prospective cohort study, we found that individuals born SGA at term exhibited poorer executive functioning than term-born non-SGA controls on performance-based measures in mid-adulthood. However, they had similar perceptions of their executive functioning as measured by self-report, except for emotional control. Moreover, the correlation between the performance-based TMT and the self-report BRIEF-A was weak in the SGA group.

## Electronic supplementary material

Below is the link to the electronic supplementary material.

Supplementary Material 1

Supplementary Material 2

Supplementary Material 3

## Data Availability

The datasets generated and/or analysed during the current study are not publicly available because permission has not been applied for from neither the participants nor the Ethical Committee, but aggregated data may be available from the corresponding author on reasonable request.
