# Supplementary material for: Executive functions in adults born small for gestational age at term: a prospective cohort study
Source: Sci Rep. 2025 Jan 29;15:3702. doi: 10.1038/s41598-025-86241-2 (PMC11779870; doi:10.1038/s41598-025-86241-2)
Supplement: Supplementary file 1 — Supplementary Material 1 [file 41598_2025_86241_MOESM1_ESM.docx]

**Table S1** Background characteristics of participants and those who did not consent to participate at 32 years

|  | **SGA** | | | | | | | **Control** | | | | | | |
| --- | --- | --- | --- | --- | --- | --- | --- | --- | --- | --- | --- | --- | --- | --- |
|  |  | Participants | |  | Non-participants | |  |  | Participants | |  | Non-participants | |  |
|  | *n* | Mean | (SD) | *n* | Mean | (SD) | *p-*value | *n* | Mean | (SD) | *n* | Mean | (SD) | *p-*value |
| Gestational age (weeks) | 56 | 39.7 | (1.2) | 30 | 39.5 | (1.3) | 0.301 | 68 | 39.8 | (1.2) | 36 | 39.4 | (1.3) | 0.095 |
| Birth weight (g) | 56 | 2916 | (205) | 30 | 2926 | (259) | 0.885 | 68 | 3695 | (459) | 36 | 3710 | (430) | 0.930 |
| Birth length (cm) | 49 | 48.5 | (1.9) | 23 | 48.3 | (1.8) | 0.604 | 65 | 51.1 | (1.9) | 35 | 50.7 | (1.6) | 0.490 |
| Ponderal index (g/cm^3^) | 49 | 2.6 | (0.2) | 23 | 2.6 | (0.2) | 0.985 | 65 | 2.8 | (0.3) | 35 | 2.8 | (0.2) | 0.461 |
| Birth head circumference (cm) | 50 | 33.9 | (1.1) | 22 | 34.1 | (1.1) | 0.707 | 64 | 35.4 | (1.2) | 35 | 35.3 | (1.1) | 0.865 |
| Maternal age at delivery (years) | 50 | 28.2 | (3.2) | 23 | 29.3 | (5.0) | 0.620 | 66 | 30.7 | (4.3) | 34 | 29.6 | (4.4) | 0.151 |
| Parental SES | 47 | 3.5 | (1.2) | 12 | 3.3 | (1.6) | 0.624 | 57 | 3.7 | (1.1) | 21 | 3.9 | (1.0) | 0.621 |
|  |  | *n* | (%) |  | *n* | (%) |  |  | *n* | (%) |  | *n* | (%) |  |
| Female |  | 31 | (55.4) |  | 13 | (43.3) | 0.288 |  | 39 | (57.4) |  | 17 | (47.2) | 0.324 |

*SD* standard deviation; *SES* socioeconomic status, *SGA* small for gestational age.
