# Supplementary material for: Executive functions in adults born small for gestational age at term: a prospective cohort study
Source: Sci Rep. 2025 Jan 29;15:3702. doi: 10.1038/s41598-025-86241-2 (PMC11779870; doi:10.1038/s41598-025-86241-2)
Supplement: Supplementary file 2 — Supplementary Material 2 [file 41598_2025_86241_MOESM2_ESM.docx]

**Table S2** Partial correlation between BRIEF-A clinical scales and composite scores and TMT scaled scores in the SGA group at 32 years of age, adjusted for sex

|  | **TMT 1** | | **TMT 2** | | **TMT 3** | | **TMT 4** | | **TMT 4-2** | |
| --- | --- | --- | --- | --- | --- | --- | --- | --- | --- | --- |
|  | *r* | *p-*value | *r* | *p-*value | *r* | *p-*value | *r* | *p-*value | *r* | *p-*value |
| Inhibit | 0.181 | 0.264 | 0.201 | 0.214 | 0.217 | 0.178 | -0.197 | 0.223 | -0.303 | 0.057 |
| Shift | -0.036 | 0.827 | -0.067 | 0.682 | 0.167 | 0.304 | -0.168 | 0.300 | -0.073 | 0.655 |
| Emotional Control | -0.053 | 0.744 | 0.070 | 0.666 | 0.265 | 0.098 | -0.303 | 0.057 | -0.280 | 0.080 |
| Self-Monitor | -0.043 | 0.791 | 0.231 | 0.151 | 0.292 | 0.068 | 0.026 | 0.876 | -0.161 | 0.320 |
| Initiate | -0.086 | 0.599 | -0.056 | 0.731 | 0.124 | 0.446 | -0.079 | 0.628 | -0.015 | 0.927 |
| Working Memory | 0.017 | 0.916 | 0.037 | 0.819 | 0.273 | 0.089 | -0.065 | 0.690 | -0.078 | 0.634 |
| Plan/Organize | 0.012 | 0.942 | -0.005 | 0.976 | 0.233 | 0.149 | -0.241 | 0.134 | -0.175 | 0.279 |
| Task Monitor | 0.206 | 0.203 | 0.101 | 0.536 | 0.327 | 0.039 | -0.186 | 0.250 | -0.217 | 0.178 |
| Organization of Materials | -0.030 | 0.855 | -0.049 | 0.766 | 0.154 | 0.343 | -0.012 | 0.940 | 0.029 | 0.860 |
| Behavioral Regulation Index | 0.020 | 0.904 | 0.127 | 0.434 | 0.282 | 0.078 | -0.220 | 0.173 | -0.263 | 0.101 |
| Metacognition Index | 0.011 | 0.946 | -0.001 | 0.993 | 0.253 | 0.116 | -0.138 | 0.395 | -0.102 | 0.533 |
| Global Executive Composite | 0.015 | 0.926 | 0.052 | 0.751 | 0.277 | 0.084 | -0.179 | 0.270 | -0.173 | 0.285 |

*BRIEF-A* Behavior Rating Inventory of Executive Function – Adult Version, *r* correlation coefficient, *SGA* small for gestational age, *TMT* Trail Making Test.
