# Supplementary material for: Executive functions in adults born small for gestational age at term: a prospective cohort study
Source: Sci Rep. 2025 Jan 29;15:3702. doi: 10.1038/s41598-025-86241-2 (PMC11779870; doi:10.1038/s41598-025-86241-2)
Supplement: Supplementary file 3 — Supplementary Material 3 [file 41598_2025_86241_MOESM3_ESM.docx]

**Table S3** Partial correlation between BRIEF-A clinical scales and composite scores and TMT scaled scores in the non-SGA control group at 32 years of age, adjusted for sex

|  | **TMT 1** | | **TMT 2** | | **TMT 3** | | **TMT 4** | | **TMT 4-2** | |
| --- | --- | --- | --- | --- | --- | --- | --- | --- | --- | --- |
|  | *r* | *p-*value | *r* | *p-*value | *r* | *p-*value | *r* | *p-*value | *r* | *p-*value |
| Inhibit | -0.012 | 0.929 | -0.130 | 0.339 | -0.030 | 0.826 | -0.239 | 0.076 | -0.081 | 0.555 |
| Shift | 0.012 | 0.928 | 0.097 | 0.479 | 0.007 | 0.961 | -0.245 | 0.069 | -0.296 | 0.027 |
| Emotional Control | -0.016 | 0.909 | 0.074 | 0.587 | 0.017 | 0.902 | -0.235 | 0.082 | -0.267 | 0.047 |
| Self-Monitor | 0.024 | 0.858 | 0.078 | 0.570 | -0.104 | 0.446 | -0.149 | 0.273 | -0.198 | 0.144 |
| Initiate | -0.072 | 0.600 | 0.012 | 0.932 | -0.093 | 0.496 | -0.067 | 0.622 | -0.068 | 0.621 |
| Working Memory | -0.057 | 0.678 | 0.066 | 0.627 | 0.034 | 0.803 | -0.166 | 0.222 | -0.201 | 0.137 |
| Plan/Organize | -0.072 | 0.597 | 0.048 | 0.727 | -0.100 | 0.462 | -0.224 | 0.098 | -0.233 | 0.084 |
| Task Monitor | -0.079 | 0.565 | 0.059 | 0.665 | -0.117 | 0.389 | -0.185 | 0.173 | -0.210 | 0.120 |
| Organization of Materials | -0.128 | 0.347 | -0.067 | 0.625 | -0.097 | 0.477 | -0.149 | 0.274 | -0.063 | 0.644 |
| Behavioral Regulation Index | -0.002 | 0.988 | 0.038 | 0.782 | -0.019 | 0.891 | -0.269 | 0.045 | -0.261 | 0.052 |
| Metacognition Index | -0.089 | 0.513 | 0.025 | 0.854 | -0.081 | 0.551 | -0.176 | 0.195 | -0.171 | 0.207 |
| Global Executive Composite | -0.062 | 0.651 | 0.031 | 0.818 | -0.062 | 0.650 | -0.219 | 0.106 | -0.213 | 0.115 |

*BRIEF-A* Behavior Rating Inventory of Executive Function – Adult Version, *r* correlation coefficient, *SGA* small for gestational age, *TMT* Trail Making Test.
